# Anosmie sans agueusie chez des patients COVID-19: à propos de 2 cas

**DOI:** 10.11604/pamj.2020.36.176.24027

**Published:** 2020-07-13

**Authors:** Abdoulaye Keita, Hamza Abdou Bacharou, Ibrahima Diallo, Alseny Camara

**Affiliations:** 1 Service Oto-Rhino-Laryngologie et Chirurgie Cervico-Faciale, Hôpital National Donka, CHU de Conakry, Guinée,; 2 Service Oto-Rhino-Laryngologie et Chirurgie Cervico-Faciale, CHU Yalgado Ouédraogo, Burkina Faso

**Keywords:** Anosmie, agueusie, COVID-19, suivi oto-rhino-laryngologique, Anosmia, aguesia, COVID-19, follow-up in ENT

## Abstract

Dans la pandémie actuelle, l'anosmie associée ou non à une agueusie semble être un symptôme fréquent en cas d'infection par le SRAS-CoV-2 responsable de COVID-19. Nous rapportons deux cas d´anosmies sans agueusies chez des patients COVID-19 adultes. L´installation a été brutale et a persisté quelques semaines après leur guérison. Ils consultèrent dans notre service ORL où ils bénéficièrent d´une abstention thérapeutique et d´une surveillance en ambulatoire. Au bout de 5 semaines, ils ont déclaré avoir récupéré leur odorat. Nous avons constaté que l´anosmie peut exister sans agueusie et persister après la guérison au COVID-19. La récupération de l´odorat est possible au bout de quelques semaines sans traitement médical. C´est pourquoi, nous recommandons un suivi des malades guéris du COVID-19 pour mieux appréhender les symptômes persistants.

## Abstract (en)

During the ongoing pandemic, anosmia, whether or not associated with aguesia, has been a common symptom in patients with SARS-CoV-2 infection causing COVID-19. We here report two cases of anosemia without aguesia in adults with COVID-19. The onset was brutal and symptomsa persisted for a few weeks after healing. The patients presented to the ENT Department where they received no therapy and underwent outpatient surveillance. After 5 weeks, patients reported they had recovered the sense of smell. This study highlights that anosmia can occur without aguesia and persist after healing in COVID-19 patients. Recovery of the smell is possible after a few weeks without medical treatment. That is why, patients follow-up is essential in subjects recovered from COVID-19 to better understand the course of persistent symptoms.

## Introduction

Dans la pandémie actuelle, l'anosmie associée ou non à une agueusie semble être un symptôme fréquent en cas d'infection par le SRAS-CoV-2 responsable de COVID-19. Elle peut être le symptôme initial de la maladie ou rester isolée chez les patients pauci-symptomatiques [1]. Ces symptômes peuvent être liés à la propension neuro-invasive du SRAS-COV-2 et à la présentation inhabituelle des manifestations de la maladie COVID-19. La description de l'anosmie et d'autres symptômes ORL est rare avec COVID-19 [2]. Notre but est de partager notre expérience par rapport à ces deux cas d´anosmies dans le contexte de COVID-19.

## Patient et observation

**Observation 1:** il s´agit d´un patient de 51 ans, médecin, résidant en milieu urbain, sans antécédent particulier. Le début de sa symptomatologie remonterait au 14 avril 2020, marquée par l´installation d´une fièvre, des céphalées, des arthralgies. Il consulta dans une formation sanitaire où il bénéficia du paracétamol et de l´azithromycine. Au bout de 2 jours, il nota la régression de symptômes sus-cités et a été testé positif au COVID-19. Au quatrième jour, il débuta le traitement par la chloroquine, vu l´installation de l´asthénie, de l´anorexie, et un prurit nasal. Ensuite, au dixième jour, il ressenti l´installation brutale d´une anosmie sans agueusie accompagnée d´une légère détresse respiratoire qui a duré environ 3h du temps. Deux jours plus tard, il nota la régression de l´anosmie. Après 2 semaines de traitement, le test au COVID-19 s´est révélé négatif. Quelques temps après sa sortie, Il consulta dans le service ORL pour anosmie.

L´examen général a noté un bon état général, sans détresse respiratoire, apyrétique. L´examen de la sphère ORL a mis en évidence une hypertrophie des cornets inférieurs avec une muqueuse nasale légèrement inflammatoire et il avait une légère perception des odeurs du parfum. Sous simple surveillance de l´anosmie, le patient rapporta un retour à la normal de son odorat au bout de 5 semaines.

**Observation 2:** patient de 59 ans, Fonctionnaire, résidant en milieu urbain, sans antécédent particulier. Il rapporte avoir développé une fièvre, une otalgie gauche, une toux et une asthénie physique. Après conseils de son frère médecin joint par téléphone, Il a reçu comme conseils de contacter le centre chargé de la gestion de la pandémie du COVID-19 où il réalisa le test qui s´est révélé positif. C´est ainsi, qu´il fut hospitalisé et soumis au protocole de la chloroquine et de l´azithromycine. L´évolution fut marquée au 5^e^jour par une intensification de l´asthénie physique et sensation d´une perte de l´odorat d´installation brutale. Par contre au 10^e^jour, il note la régression de la symptomatologie. Le patient fut déclaré guéri au 16^e^jour après réalisation des tests de contrôle. Il fut orienté vers un service d´ORL pour le suivi de son anosmie.

L´examen général notait un bon état général, apyrétique, sans dyspnée avec un état hémodynamique stable. L´examen ORL notait une hypertrophie des cornets inférieurs et muqueuse nasale violacée. Son médecin traitant a opté pour une abstention thérapeutique. L´évolution a été marquée par la disparition de l´anosmie au bout de 4 semaines.

## Discussion

L'anosmie et/ou l'agueusie en l'absence d'autres maladies respiratoires telles que la rhinite allergique, la rhinosinusite aiguë ou la rhinosinusite chronique, devraient alerter les médecins sur la possibilité d'une infection par COVID-19 [3] et inciter sérieusement à s'auto-isoler et à tester ces personnes [3]. Ça été le cas chez nos patients qui ont été testé positif au COVID-19 et c´est au cours de leur convalescence que l´anosmie sans agueusie s´est installée brutalement, chose qui a été décrit dans certaines études [4]. Cependant, chez les patients COVID-19, il ne semble pas y avoir une composante inflammatoire aussi importante et l'altération de l'odorat n'est généralement pas accompagnée de symptômes de rhinite [1, 2]. Par conséquent, une hypothèse pourrait être que les altérations sont dues à des dommages causés par le virus aux voies olfactives [5]. L'anosmie a déjà été décrite dans les infections à coronavirus courantes [6].

Récemment, une probabilité d'association entre COVID-19 et une fonction olfactive altérée a été signalée en Corée du Sud, en Iran, en Italie, en France, au Royaume-Uni et aux États-Unis [4, 5]. Cependant, à notre connaissance, l'association définitive entre COVID-19 et l'anosmie n'a pas été publiée depuis de la pandémie [7]. Mais de nos jours, Il existe des preuves anecdotiques accumulées que l'anosmie et/ou l´agueusie sont associées à la pandémie de COVID-19 [2]. L'atteinte olfactive auto déclarée a récemment été reconnue comme une caractéristique de COVID-19 et peut être un prédicteur important des résultats cliniques [8]. Les cas d´anosmie chez nos patients se sont auto-découvert tout en précisant l´absence de l´agueusie. Liang En Wee, dans une cohorte multicentrique de patients COVID-19, a rapporté que 85,6% avaient des troubles olfactifs ou gustatifs [9].

Selon la gravité de l'infection initiale, l´odorat peut revenir en quelques jours ou semaines [3]. La plupart des patients souffrant d'anosmie ou d'agueusie ont récupéré en 3 semaines. Le délai médian de récupération était de 7 jours pour les deux symptômes selon Lee Y. dans sa série [10]. Par contre, étant donné qu´ils étaient suivis en ambulatoire dans le service, nos patients ont affirmé avoir récupéré leur odorat au bout de 5 semaines pour le premier cas et 4 semaines pour le second. A comparer avec les auteurs, nous en déduisons que l´évaluation de la récupération de l´anosmie des patients COVID-19 est patient et/ou examinateur dépendant.

## Conclusion

L´anosmie est un symptôme observé chez les patients COVID-19. Il persiste généralement quelques semaines après la guérison du patient du COVID-19. La disparition de l´anosmie est fonction d´un patient à un autre. A la lumière de ce qui précède, nous trouvons opportun d´envisager le suivi des patients COVID-19 déclarés guéris ayant des symptômes persistants notamment l´anosmie et/ou l´agueusie. En tenant compte du domaine médical approprié.

## References

[1] Reinhard A, Ikonomidis C, Broome M, Gorostidi F (2020). Anosmia and COVID-19. Rev Med Suisse.

[2] Klopfenstein T, Kadiane-Oussou NJ, Toko L, Royer P-Y, Lepiller Q, Gendrin V (2020). Features of anosmia in COVID-19. Med Mal Infect.

[3] Lorenzo Villalba N, Maouche Y, Alonso Ortiz MB, Cordoba Sosa Z, Chahbazian JB, Syrovatkova A (2020). Anosmia and Dysgeusia in the absence of other respiratory diseases: should COVID-19 infection be considered?. Eur J Case Rep Intern Med.

[4] Gilani S, Roditi R, Naraghi M (2020). COVID-19 and anosmia in Tehran, Iran. Medical Hypotheses.

[5] Brann D, Tsukahara T, Weinreb C, Logan DW, Datta SR (2020). Non-neural expression of SARS-CoV-2 entry genes in the olfactory epithelium suggests mechanisms underlying anosmia in COVID-19 patients. bioRxiv.

[6] Hopkins C, Surda P, Kumar N (2020). Presentation of new onset anosmia during the COVID-19 pandemic. Rhinology.

[7] Heidari F, Karimi E, Firouzifar M, Khamushian P, Ansari R, Ardehali MM (2020). Anosmia as a prominent symptom of COVID-19 infection. Rhinology.

[8] Yan CH, Faraji F, Prajapati DP, Ostrander BT, DeConde AS (2020). Self-reported olfactory loss associates with outpatient clinical course in Covid-19. International Forum of Allergy & Rhinology.

[9] Wee LE, Chan YFZ, Teo NWY, Cherng BPZ, Thien SY, Wong HM (2020). The role of self-reported olfactory and gustatory dysfunction as a screening criterion for suspected COVID-1 Eur Arch Otorhinolaryngol.

[10] Lee Y, Min P, Lee S, Kim S-W (2020). Prevalence and Duration of Acute Loss of Smell or Taste in COVID-19 Patients. J Korean Med Sci.

